# Final Report on Clinical Outcomes and Tumor Recurrence Patterns of a Pilot Study Assessing Efficacy of Belinostat (PXD-101) with Chemoradiation for Newly Diagnosed Glioblastoma

**DOI:** 10.3390/tomography8020057

**Published:** 2022-03-03

**Authors:** Karen Xu, Karthik Ramesh, Vicki Huang, Saumya S. Gurbani, James Scott Cordova, Eduard Schreibmann, Brent D. Weinberg, Soma Sengupta, Alfredo D. Voloschin, Matthias Holdhoff, Peter B. Barker, Lawrence R. Kleinberg, Jeffrey J. Olson, Hui-Kuo G. Shu, Hyunsuk Shim

**Affiliations:** 1 Department of Radiation Oncology, Emory University, Atlanta, GA 30322, USA; kxu@sbrmc.org (K.X.); karthik.ramesh@emory.edu (K.R.); vicki.huang@emory.edu (V.H.); saumyasg@gmail.com (S.S.G.); jscordova@wustl.edu (J.S.C.); eschre2@emory.edu (E.S.); 2 Department of Biomedical Engineering, Georgia Institute of Technology, Atlanta, GA 30332, USA; 3 Department of Radiology and Imaging Sciences, Emory University, Atlanta, GA 30322, USA; brent.d.weinberg@emory.edu; 4 Winship Cancer Institute, Emory University, Atlanta, GA 30322, USA; jolson@emory.edu; 5 Department of Hematology and Medical Oncology, Emory University, Atlanta, GA 30322, USA; sengupsm@ucmail.uc.edu (S.S.); alfredo.voloschin@emory.edu (A.D.V.); 6 Department of Oncology, Johns Hopkins University, Baltimore, MD 21218, USA; mholdho1@jhmi.edu; 7 Department of Radiology and Radiological Science, Johns Hopkins University, Baltimore, MD 21205, USA; pbarker2@jhmi.edu; 8 Department of Radiation Oncology, Johns Hopkins University, Baltimore, MD 21218, USA; kleinla@jhmi.edu; 9 Department of Neurosurgery, Emory University, Atlanta, GA 30322, USA

**Keywords:** glioblastoma, histone deacetylase, epigenetic drug, radiation sensitizer, magnetic resonance spectroscopy

## Abstract

Glioblastoma (GBM) is highly aggressive and has a poor prognosis. Belinostat is a histone deacetylase inhibitor with blood–brain barrier permeability, anti-GBM activity, and the potential to enhance chemoradiation. The purpose of this clinical trial was to assess the efficacy of combining belinostat with standard-of-care therapy. Thirteen patients were enrolled in each of control and belinostat cohorts. The belinostat cohort was given a belinostat regimen (500–750 mg/m^2^ 1×/day × 5 days) every three weeks (weeks 0, 3, and 6 of RT). All patients received temozolomide and radiation therapy (RT). RT margins of 5–10 mm were added to generate clinical tumor volumes and 3 mm added to create planning target volumes. Median overall survival (OS) was 15.8 months for the control cohort and 18.5 months for the belinostat cohort (*p* = 0.53). The recurrence volumes (rGTVs) for the control cohort occurred in areas that received higher radiation doses than that in the belinostat cohort. For those belinostat patients who experienced out-of-field recurrence, tumors were detectable by spectroscopic MRI before RT. Recurrence analysis suggests better in-field control with belinostat. This study highlights the potential of belinostat as a synergistic therapeutic agent for GBM. It may be particularly beneficial to combine this radio-sensitizing effect with spectroscopic MRI-guided RT.

## 1. Introduction

Glioblastoma (GBM) is the most common primary brain tumor in adults. It is highly aggressive and associated with poor prognosis (despite multimodal treatment). The current treatment paradigm consists of maximal safe neurosurgical resection followed by radiation therapy (RT) with concurrent and adjuvant temozolomide (TMZ). This regimen consists of focal RT to 60 Gy over 6 weeks with concurrent TMZ given at 75 mg/m^2^/day followed by adjuvant TMZ given at 150–200 mg/m^2^/day for days 1 to 5 of a 28-day cycle for up to 12 months. Despite comprehensive treatments, median overall survival (OS) remains at 16 months [1,2,3,4,5,6]. 

Histone deacetylases regulate a wide variety of cellular functions and play a role in re-differentiation of various tumors. Histone deacetylase inhibitors (HDACi) have been shown to improve outcomes for patients with gliomas [7]. In 2006, suberanilohydroxamic acid (SAHA), a first-generation HDACi which targets multiple class I and class II HDAC family members, became the first HDACi to receive FDA approval for advanced cutaneous T-cell lymphomas [8].

Belinostat (PXD-101, Acrotech Biopharma, LLC, East Windsor, NJ), a later generation pan-HDACi, improves upon SAHA with increased potency and BBB penetration [9,10]. Belinostat received FDA approval for patients with relapsed/refractory peripheral T-cell lymphoma in 2014 [11]. Preclinical investigations have shown antitumor effects in orthotopic glioma animal models [12]. This suggests that development of a potent HDACi capable of penetrating the BBB has the potential to improve outcomes of patients with GBM. In addition to anti-tumor effects, we reported that belinostat restores the bottle neck enzyme levels of normal brain metabolites, N-acetylaspartate (NAA) and myo-inositol, in vitro to a greater extent than other HDACis [12]. Furthermore, spectroscopic magnetic resonance imaging (sMRI), a quantitative imaging technique that assesses metabolic responses in vivo without any contrast agent injection, has been used to show a GBM patient with an IDH mutation (without MGMT promoter hypermethylation) exhibited remarkable response to belinostat combined with chemoradiation therapy [13]. In that case restoration of NAA, creatine and myo-inositol, reached the levels of healthy subjects.

We previously reported interim PFS and sMRI findings in GBM patients receiving belinostat [12,13]. With 50 months of median follow-up, we report the final clinical outcomes and tumor recurrence patterns of patients enrolled in this study. 

## 2. Materials and Methods

Patients with newly diagnosed GBM were enrolled in either the control or treatment arm of the Institutional Review Board (IRB)-approved clinical trial at Emory and Johns Hopkins (ClinicalTrials.gov ID NCT02137759, accessed on 26 April 2021). Patients on the treatment arm received intravenous belinostat (Acrotech Biopharma, LLC, East Windsor, NJ, USA) as an investigational therapeutic. 

This was not a randomized study; patients were serially enrolled into the control arm from 2014 to 2015 and then the belinostat treatment arm from 2015 to 2018. All patients, if surgical candidates, underwent maximal safe tumor resection before enrolling in the study. Patients in both arms received standard treatment including daily TMZ (75 mg/m^2^) and focal radiation doses of 51 Gy to T2/FLAIR abnormality (GTV1) and 60 Gy to the resection cavity/residual contrast-enhancing (CE) tissue (GTV2) on T1-weighted contrast-enhanced MRI (T1w-CE). Margins of 5 to 10 mm were added to generate clinical tumor volumes (CTVs) and 3 to 5 mm to generate planning treatment volumes (PTVs) to account for microscopic disease spread and treatment setup uncertainty, respectively. PTV1 is the FLAIR abnormal volume receiving 51 Gy while PTV2 is the T1w-CE volume receiving 60 Gy. The choice to use two radiation target volumes is common practice for participating institutions in this trial and recognized by organizations such as RTOG and ASCO [14,15]. Further, a two-target regimen has been conducted in other clinical trials to success [3,14,15,16,17,18]. Enhancing lesions in T1w-CE imaging are highly specific for tumor as those regions of the brain have experienced a breakdown of the BBB, justifying a higher radiation dose of 60 Gy. While spreading hyperintensities in T2/FLAIR are indicative of infiltrating tumor, they also include edema and inflammation, justifying a lower radiation dose. 

**Figure 1:**
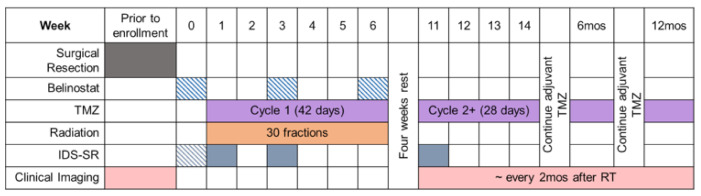
One-year timeline of chemotherapy, intravenous belinostat, and radiation for patients in NCT02137759.

In the treatment arm, patients received daily intravenous doses of belinostat for five consecutive days in three cycles, three weeks apart, beginning one week before the start of chemoradiation. The first three patients received 750 mg/m^2^ of belinostat. However, due to two of the patients having adverse hematologic toxicity during the course of belinostat, TMZ, and radiation, the belinostat dose was lowered to 500 mg/m^2^ for the remaining patients in the trial. Patients were followed with standard MRIs for 12 months post-treatment or until radiographic progression of disease. The study timeline for the belinostat cohort is summarized in Figure 1. Patients in both the control and belinostat cohorts underwent the same radiation therapy dose plan guided by T1w-CE and T2/FLAIR imaging. PFS is reported for patients based on time to radiologic confirmation of disease progression from the date of surgery. Data are right censored for patients who were lost to follow-up. Kaplan-Meier curves for OS were generated.

sMRI data were acquired for patients before starting treatment and four weeks post-RT. The sMRI data acquisition combined 3D echo-planar spectroscopic imaging, with GRAPPA (Generalized Autocalibrating Partial Parallel Acquisition) parallelization, and elliptical k-space encoding (TE/TR/FA = 17.6ms/1551ms/71°) on 3T MRI scanners with a 32-channel head coil array (Siemens Healthineers or Philips Healthcare) [19]. Raw data were processed using the Metabolite Imaging and Data Analysis System (MIDAS) (University of Miami) [19,20,21] with a nominal voxel size of 4.4 × 4.4 × 5.6 mm. Metabolite maps generated included choline (Cho), Creatine (Cr), N-acetylaspartate (NAA), as well as Cho/NAA ratio maps [22]. Metabolite and ratio maps were then imported into the Brain Imaging Collaboration Suite (BrICS) for registration with the radiation planning MRI images [23]. A new volume was created based on union of Cho/NAA elevated lesions with residual contrast-enhancing volume (excluding resection cavity) as described in our dose escalation trial scheme [23].

After completing enrollment in August of 2018, we continued to track patients to assess long-term outcomes and recurrence patterns. Tumor progression was determined using a combination of the BT-RADS (The Brain Tumor Reporting and Data System) structured reporting criteria as well as clinical judgment, which is standard practice at Emory University [24,25,26,27]. For each patient, T1w-CE MRIs were acquired at the radiologically confirmed progression dates and co-registered to MRIs used for RT planning. The recurrence volumes based on enhancement in T1w-CE MRIs (rGTVs) were generated by manually contouring abnormal enhancement in the progressed MRIs. Overlap statistics were calculated between rGTV and PTVs (PTV 1 and 2) as well as calculation of the minimum, maximum, and mean radiation doses that the rGTV volume received using in-house automated algorithms to perform voxel-wise dose comparisons. Radiation doses received by each volume were extracted from the clinical system using the Eclipse Scripting Application Programming Interface (ESAPI) which is built into the Eclipse treatment-planning platform (Varian Medical Systems, Inc., Palo Alto, CA, USA). Incorporation with this platform enabled automation of our method, saving time and errors that often occur from manual data extraction, with the additional benefit of using the original measurements of the radiation dose clouds for a comparison to tumor recurrence in terms of the range and median doses received by those regions. Two patients were excluded from analysis of rGTV due to follow-up imaging being unavailable.

## 3. Results

A total of 26 patients were enrolled (13 control and 13 belinostat) with median follow-up of 50 months. Patient/tumor characteristics were similar between cohorts (Table A1); summary statistics are highlighted in Table 1. 

**Figure 2:**
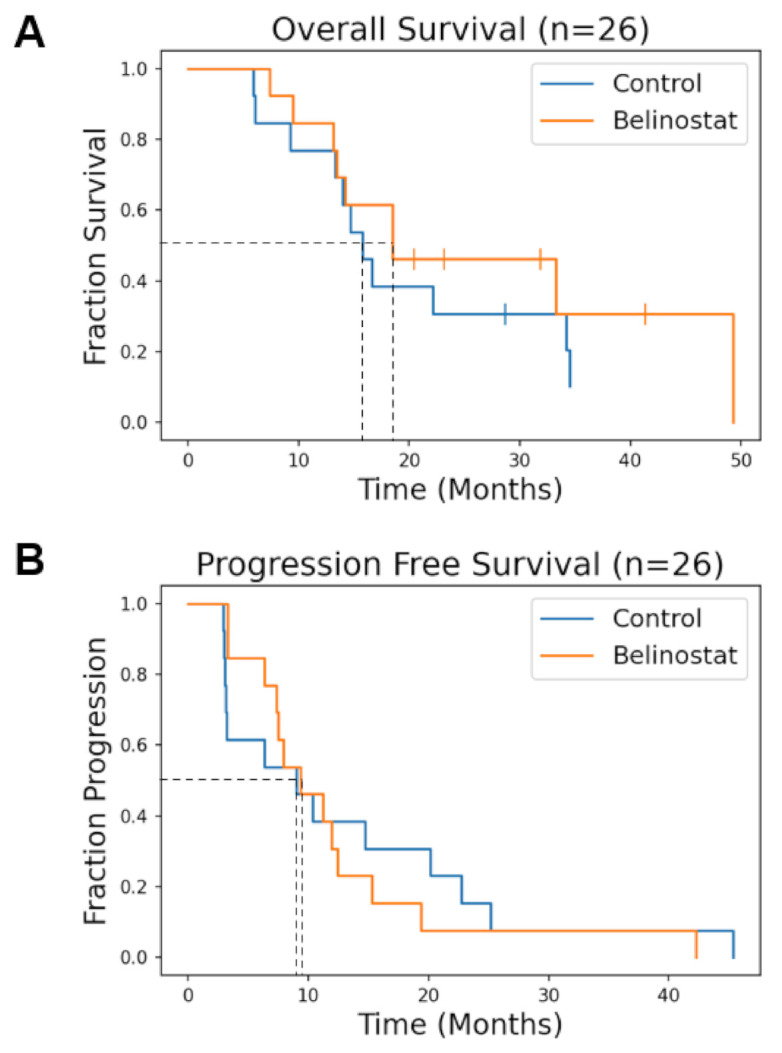
(**A**) Overall Survival (OS) Kaplan-Meier Survival Curves. The median OS for the control and belinostat cohorts was 15.8 and 18.5 months, respectively (*p* = 0.53), for all patients. (**B**) Progression free survival (PFS) for all patients. The median PFS for control and belinostat cohorts was 9.0 months and 9.3 months, respectively (*p* = 0.75).

Median OS was 15.8 months for the control cohort and 18.5 months for the belinostat cohort (*p* = 0.53). Comparison of OS curves for the two cohorts are shown in Figure 2A. 6-month PFS was 54% and 84% (*p* = 0.073) for the control and the belinostat cohorts, respectively, reported previously [12]. The median PFS was 9.0 months for the control and 9.3 months for the belinostat cohorts (*p* = 0.75). 

**Figure 3:**
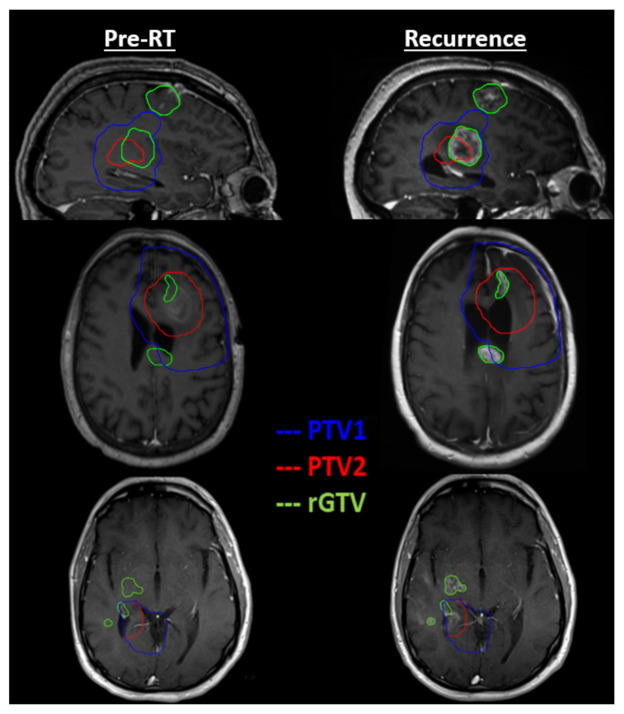
T1 post-contrast imaging in three patients, by horizontal row, with out of field recurrence in cohort 2. For each patient, the enhancing recurrence contour (rGTV) encompasses lesion that has spread outside the extent of radiation treatment targets PTV1 (guided by T2/FLAIR) or PTV2 (guided by T1w-CE).

To gain a greater understanding of enhancing recurrence patterns relative the radiation dose distribution in study patients, we determined the actual dose received by the region of recurrence. The minimum, maximum, and mean radiation dose to rGTV, the percentage of rGTV that occurred in PTV1, and the percentage of rGTV that occurred in PTV2 for each patient enrolled in the trial was determined and is listed in Table 2. For example, for patient QINU01EM008, the minimum, maximum, and mean radiation dose to rGTV was 11.8 Gy, 64.7 Gy, and 58.7 Gy, respectively. For that same patient, 90.9% of the rGTV recurrence volume occurred within the PTV1 treatment contour while 69.9% of the recurrence occurred within PTV2. This would suggest that for this patient, most of the recurred lesion was within the PTV radiation targets. For the control versus the belinostat cohort, the minimum, maximum, and mean radiation dose to rGTV was on average 54.6 versus 45.0 Gy (*p* = 0.20), 64.1 versus 57.9 Gy (*p* = 0.11), and 62.0 versus 51.5 Gy (*p* = 0.042), respectively. For the control versus the belinostat cohorts, the mean percentage of rGTV within PTV1 and PTV2 was 99.3% versus 73.9% (*p* = 0.052) and 97.1% versus 69.0% (*p* = 0.034), respectively. While all control patients had in-field failure as the site of first recurrence, only ten out of 13 belinostat patients failed in-field initially. Figure 3 illustrates out-of-field failure in the three remaining belinostat patients (cohort 2). For those patients, there were regions of rGTV that fell squarely within the PTV1 and PTV2 contours, illustrating instances of failure “in-field” of radiation. However, these three cases also show instances where rGTV was either completely outside of the PTV contours or spread from outside of the PTV contours (as suggested by the red arrows).

**Figure 4:**
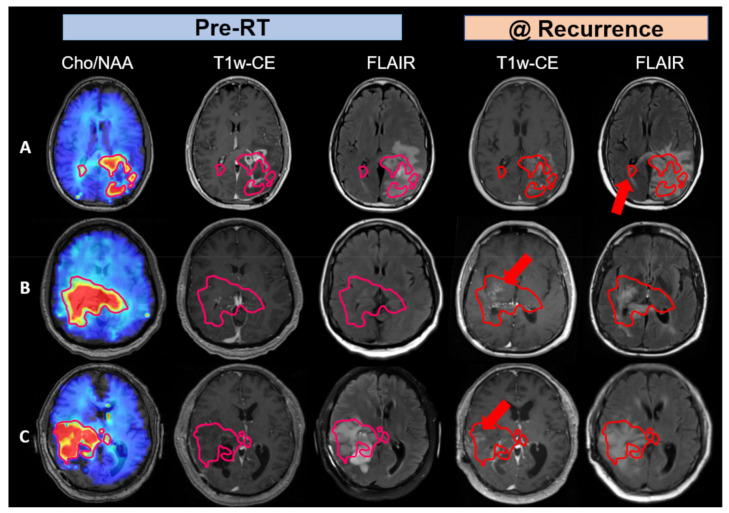
Pre-RT sMRI scans suggest out-of-field recurrence in the belinostat cohort with tumor infiltration beyond what is shown in standard imaging (indicated by red arrows). (**A**) This patient had two-fold Cho/NAA elevation that had spread contralaterally. While pre-RT standard imaging failed to detect this, FLAIR hyperintensity at recurrence mimics the direction of tumor infiltration detected by pre-RT sMRI. (**B**) The second patient had tumor infiltration across the midline that wasn’t detected by pre-RT standard imaging. Lesions at recurrence in both T1w-CE and FLAIR MRIs confirm Cho/NAA abnormalities in the pre-RT sMRI scan, suggesting standard imaging underestimated the extent of tumor infiltration. (**C**) This patient had a large lesion of metabolically active tumor that was not shown in T1w-CE, thus, undertreated, which became apparent at recurrence.

In Figure 4, we sought to explore why three patients in the belinostat cohort had worse outcomes than historical controls. We investigated Cho/NAA abnormalities in these patients’ pre-RT sMRI scan as Cho/NAA is a sensitive marker for regions at risk of recurrence [22]. The contours in the first column of Figure 4 depict the margin of two-fold or greater elevations in the Cho/NAA ratio (compared to the contralateral normal-appearing white matter). In the second column these are overlaid on the standard contrast enhanced MRI images to show the substantial difference between what is usually recognized as residual tumor and regions that predict tumor extent by Cho/NAA. This level of Cho/NAA elevation equates to a mean Z-score of 6.62 as reported previously (with >99.999% confidence) [22]. In the third column, the margins for the two-fold or greater elevations in the Cho/NAA ratio are overlaid on FLAIR images. This shows somewhat greater but still incomplete concordance with the extent of the tumor predicted by the Cho/NAA ratio. 

Axial slices in Figure 4 were chosen to most emphasize regions of tumor recurrence that were detectable in pre-RT Cho/NAA imaging. In row 4A, Cho/NAA elevation showed tumor spreading to the contralateral side through the corpus callosum, which wasn’t apparent in pre-RT T1w-CE or FLAIR MRIs, and thus was not treated within the radiation treatment plan. At recurrence, FLAIR hyperintensity had spread contralaterally in a similar direction to the Cho/NAA abnormality confirming tumor infiltration that was not treated during RT. In row 4B, the pre-RT Cho/NAA abnormality crosses the midline, which wasn’t apparent in T1w-CE or even in FLAIR MRIs. Less than a year after RT, recurrence patterns with standard imaging confirmed findings in the pre-RT sMRI scan with both enhancement and FLAIR hyperintensity apparent within the pre-RT Cho/NAA contour. For this patient especially, the morphology of the T1w-CE and FLAIR lesions evolved throughout the follow-up period to approximate the morphology of the pre-RT Cho/NAA abnormality. In row 4C, the pre-RT Cho/NAA abnormality spread widely anterior to the resection cavity, which was not visible in T1w-CE. Thus, this elevated Cho/NAA ratio abnormality was incompletely treated with high dose radiation. The recurrence pattern confirmed the findings in the pre-RT sMRI scan. The T1w-CE and FLAIR studies demonstrate increasing enhancement evolved throughout the follow-up period to approximate the morphology of the pre-RT Cho/NAA ratio abnormality. In summary, these three cases suggest that standard imaging underestimates the true extent of tumor infiltration, causing these patients to be undertreated and have a lower survival. When excluding these three patients, the median OS of the belinostat cohort increases to over 30 months. 

Treatment with belinostat given at a dose of 750 mg/m^2^/day × five days every three weeks during week zero (pre-RT) as well as week three and week six of RT resulted in dose limiting toxicities (DLTs) (two with grade 4 thrombocytopenia and one with grade neutropenia) in two of three patients. However, after de-escalation of the belinostat dose to 500 mg/m^2^/day in the remaining cohort patients, no further DLTs were experienced. Significant toxicities (grade 3 or greater) that were judged to at least possibly be due to therapy for our control and belinostat cohorts are summarized in Table 1. Overall, the addition of belinostat at the 500 mg/m^2^/day dosing to concurrent RT/TMZ appears to be well tolerated.

## 4. Discussion

Here, we report the final results of a pilot study adding belinostat to concurrent RT and TMZ for patients with newly diagnosed GBMs [12]. Previously, the cohort receiving belinostat showed an improved six-month PFS compared to the control cohort (54% vs. 84%, *p* = 0.073). In Figure 2B, the beneficial effects of belinostat delaying tumor progression appear to decline by month nine. A speculated reason for the improved PFS at six months but not by nine months is that belinostat was only given to subjects for a short duration throughout RT. HDACis such as belinostat are known to be reversible drugs, with epigenetic stress in tumors and the microenvironment resuming in the absence of HDACis resulting in a truncated effect. In our final analysis, the median overall survival was promising appearing slightly longer (by about 2.7 months) for the belinostat cohort. While the result is not statistically significant, this may be in part due to the small sample size. In a recent cooperative group trial (NRG-BN001) comparing dose-escalated to conventional dose RT for newly diagnosed GBM patients, patients on the experimental arm that received dose-escalated photon radiation had a median OS of 18.7 months [3]. The similarity in median OS benefit between the dose-escalated treatment arm of BN001 and our belinostat cohort points to the potential of belinostat possibly having a radiation sensitizer effect on patients comparable to dose escalation. In our trial, the voxel-based analysis of recurrence patterns comparing recurrent voxels to those in PTV2 indicates a potential shift of recurrences being more out-of-field suggesting that in-field control was improved due to the radiosensitizing effects of belinostat. 

Pre-RT sMRI scans for a few representative belinostat patients reveal larger tumor extents (median volume difference was 6.2-folds between union of Cho/NAA ≥ 2x and residual tumor vs. residual tumor volume in all belinostat group patients) than were detected in standard imaging. Therefore, these portions of the lesion (as defined by Cho/NAA) were left undertreated. RT plans for all patients on the trial were guided by abnormality in T1w-CE and FLAIR only. Recurrence patterns confirm that pre-RT sMRI findings provide a possible reason for some in the belinostat cohort having an OS that was lower than that in historical controls. Due to the initial study design, we were only permitted to perform sMRI scans up to four weeks after completion of radiation therapy. In the future, we hope to include the sMRI sequence during patient follow-up as it is a valuable predictor of tumor infiltration extent. 

The wide variability in OS suggests that some patients respond much more positively to a belinostat regimen while for others, the effect is minimal. While HDAC inhibitors are powerful epigenetic modulators, they are still target-specific drugs. They are hypothesized to work in the subgroup of patients whose tumorigenesis is driven by epigenetic modifications. This encourages investigation into whether certain subtypes of GBMs are more genetically predisposed to react positively to HDACi’s like belinostat. Unfortunately, our sample size was too small to identify molecular subtypes that were responsive to belinostat. Interestingly, one patient with an IDH mutation who responded remarkably well to belinostat [13]. With this patient, there was not only a visible size reduction of metabolically active tumor volume but also the metabolic activity in the contralateral side of the brain was restored to healthy levels. This supports the observations reported by other groups that IDH1/2 mutations in GBM are associated with a fascinating link to 2-hydroxyglutarate (2-HG) accumulation representing an altered metabolite profile, which may have broad implications for both cancer epigenetics and clinical management of disease [28]. While it is difficult to run a clinical trial in GBM with IDH mutation in a single institution since the incident rate is low, it may be beneficial to run a multisite trial to determine if GBM with IDH mutation are unusually sensitive to belinostat. 

Patients receiving belinostat on our study appear to have a different recurrence pattern than those in the control cohort. In particular, the mean radiation dose to the rGTV region was statistically significantly lower in the belinostat cohort than that in the control cohort (51.5 Gy vs. 62.0 Gy, *p* = 0.042) while other comparisons of the minimum and maximum dose to rGTV were similarly trending in the same direction. This suggests that concurrent belinostat may be delaying recurrences in regions that received higher radiation doses. The overlap between rGTV and PTV treatment zones was also lower in the belinostat cohort than that in the control cohort. In particular, the mean overlap between rGTV and PTV2, which received the highest dose of radiation, was significantly lower in the belinostat patients than that for the control (69.0% vs. 97.1%, *p* = 0.034). Overall, these trends look promising for belinostat’s activity in GBM especially in concert with full dose RT. This is further supported by the three patients in the belinostat cohort that had out-of-field recurrence as the first site of recurrence. In them the belinostat was unable to significantly impede recurrences when RT dose was inadequate. 

Finally, the addition of belinostat to concurrent RT/TMZ at a dose of 750 mg/m^2^/day x five days every three weeks starting one week prior to RT did result in DLTs in two of three patients that completed this therapy. In one case, grade 4 thrombocytopenia first developed just prior to cycle one of adjuvant TMZ. In the other case, grade 4 thrombocytopenia and neutropenia did not develop until after receiving three cycles of adjuvant TMZ (more than four months following the last treatment with belinostat). While it is possible that these were spurious events as these hematologic toxicities can occur with TMZ alone, the lack of hematologic DLTs in our control cohort (n = 13) and the remaining belinostat cohort (n = 10) treated at 500 mg/m^2^/day argues for implicating belinostat in these DLTs. Also, at the lower belinostat dose, no unusual toxicity was definitively noted. Thus, based on our limited experience, the dosing regimen of three cycles of belinostat at 500 mg/m^2^/day x five days every three weeks during concurrent RT/TMZ is well tolerated and should serve as a point of departure for dosing in future trials evaluating belinostat in combination with RT and TMZ. 

Due to limitations in the study including a small sample size in each cohort, a wide range of survival responses to belinostat, as well as toxicities from initially larger doses of administered belinostat, future work will require larger cohorts with manageable belinostat dosing regimens to discern the true effect size of this treatment. In this trial, belinostat was only administered during RT. Since an HDACi such as belinostat is a reversible drug and epigenetic modification-induced stress remains, we hypothesize that extended use of belinostat during the maintenance period after radiation therapy may further improve outcomes in patients. This will require future testing to assess the safety and efficacy of belinostat when combined with adjuvant TMZ. Based on the results of this pilot study, we are planning a multisite trial with a larger cohort of patients that will also including maintenance belinostat after sMRI-guided radiation treatment. 

## 5. Conclusions

In summary, we have established that belinostat can be safely given with concurrent RT and TMZ and is trending towards improving outcomes in newly diagnosed GBM patients. Since recurrence volumes in the control cohort had larger overlap with PTV2s (volume receiving 60 Gy) than in the belinostat cohort, this suggests that belinostat has a better likelihood of delaying recurrence in those regions receiving 60 Gy. Trends in this study highlight the potential of belinostat, a BBB-penetrating HDACi, as a synergistic therapeutic agent for GBM treatment.

## Figures and Tables

**Table 1:** Summary statistics for both cohorts including tabulation of significant toxicities (≥grade 3) in all patients. * All occurrences were at 750 mg/m^2^/day dosing.

	Control	Belinostat
Number of Patients	13	13
Age	58.5 ± 11.1	51.2 ± 11.6
IDH1 Mutation	1 (7.7%)	1 (7.7%)
MGMT Methylated	4 (30.8%)	4 (30.8%)
Toxicities (Grade)		
Thrombocytopenia (4)	0/13	2/13 *
Neutropenia (4)	0/13	1/13 *
Lymphopenia (3)	3/13	1/13 *
Constipation (3)	1/13	1/13
Fatigue (3)	1/13	1/13
Confusion (3)	1/13	0/13

**Table 2:** Minimum, maximum, and mean radiation dose to recurrence volume (rGTV), overlap between rGTV and PTV1, and overlap between rGTV and PTV2.

Study ID	Minimum Dose (Gy)	Maximum Dose (Gy)	Mean Dose (Gy)	rGTV Overlap with PTV1	rGTV Overlap with PTV2
Cohort 1					
QINU01EM001	59.0	65.2	62.3	100.0%	100.0%
QINU01EM002	61.1	63.4	62.4	100.0%	100.0%
QINU01EM003	59.4	63.8	61.8	100.0%	100.0%
QINU01EM004	61.9	64.5	63.1	100.0%	100.0%
QINU01EM005	60.3	63.5	62.5	100.0%	100.0%
QINU01EM006	61.1	64.2	62.5	100.0%	100.0%
QINU01EM007	60.2	63.6	62.0	100.0%	100.0%
QINU01EM008	11.8	64.7	58.7	90.9%	69.9%
QINU01EM010	52.2	64.7	62.3	100.0%	99.1%
QINU01EM011	44.1	64.7	62.2	99.8%	99.5%
QINU01EM012	58.7	63.6	61.9	100.0%	94.5%
QINU01EM013	59.0	64.1	61.8	100.0%	100.0%
QINU01JH001	60.1	63.2	61.4	100.0%	99.8%
Cohort 2					
QINU01EM014	52.2	63.2	58.2	100.0%	50.1%
QINU01EM015	58.7	62.8	61.2	100.0%	96.2%
QINU01EM016	56.2	64.9	62.8	99.2%	99.2%
QINU01EM017	10.9	50.3	25.2	0.0%	0.0%
QINU01EM019	60.2	63.2	61.6	100.0%	100.0%
QINU01EM021	*	*	*	*	*
QINU01EM022	59.6	62.8	61.0	100.0%	100.0%
QINU01EM023	45.6	53.4	50.3	0.0%	0.0%
QINU01EM024	59.5	63.8	61.6	100.0%	100.0%
QINU01JH002	60.0	62.1	61.0	100.0%	100.0%
QINU01JH003	1.9	63.8	32.3	13.4%	13.4%
QINU01EM025	19.8	21.7	20.9	100.0%	100.0%
QINU01EM026	*	*	*	*	*

* Follow-up imaging was not available for recurrence analysis.

## Data Availability

The data presented in this study are available on request from the corresponding author.

## Appendix A

**Table A1:** The characteristics and outcome for each patient enrolled in the trial.

Study ID	Age (at RT)	IDH Mutation (Yes = 1, No = 0)	MGMT Methylation (Yes = 1, No = 0)	OS (Months since Surgery)	Event = 1, Censored = 0	PFS (Months since Surgery)
COHORT 1						
QINU01EM001	61	0	-	16.6	1	6.4
QINU01EM002	45	1	1	34.2	1	20.2
QINU01EM003	55	0	0	28.7	0	25.2
QINU01EM004	60	0	1	62.2	0	45.4
QINU01EM005	71	0	1	14.7	1	9.0
QINU01EM006	82	0	0	34.5	1	22.8
QINU01EM007	45	0	0	22.1	1	14.7
QINU01EM008	40	0	0	13.3	1	3.1
QINU01EM010	61	0	0	15.8	1	3.2
QINU01EM011	60	0	1	6.1	1	3.1
QINU01EM012	63	0	0	9.2	1	2.9
QINU01EM013	51	0	0	5.9	1	3.0
QINU01JH001	67	0	0	14.0	1	10.4
COHORT 2						
QINU01EM014	58	0	1	49.3	1	42.3
QINU01EM015	52	0	0	18.5	1	7.5
QINU01EM016	50	0	-	18.5	1	9.3
QINU01EM017	44	0	-	7.4	1	3.3
QINU01EM019	27	1	0	33.3	1	19.3
QINU01EM021	68	0	0	13.4	1	3.3
QINU01EM022	66	0	1	23.1	0	12.5
QINU01EM023	61	0	1	20.5	0	11.2
QINU01EM024	30	0	0	41.4	0	7.4
QINU01JH002	55	0	0	13.1	1	7.9
QINU01JH003	52	0	1	14.2	1	11.9
QINU01EM025	50	0	0	9.4	1	6.4
QINU01EM026	53	0	0	31.9	0	15.3
